# Maintenance treatment with chemotherapy and immunotherapy in non-small cell lung cancer: a case report

**DOI:** 10.3389/fonc.2012.00152

**Published:** 2012-10-26

**Authors:** Anabella Llanos, Mariana Savignano, Gabriela Cinat

**Affiliations:** ^1^Department of Sarcoma and Melanoma, Instituto Angel H. RoffoBuenos Aires, Argentina; ^2^Department of Clinical Oncology, Instituto Angel H. RoffoBuenos Aires, Argentina

**Keywords:** vaccines, lung cancer, immunotherapy, chemotherapy, concurrent review

## Abstract

A 53-years-old woman was diagnosed with lung adenocarcinoma state IV (synchronous pleural involvement) in April 2009. First-line systemic treatment included six cycles of Carboplatin, Paclitaxel, and Bevacizumab. Partial response was achieved. Maintenance therapy with Bevacizumab and Pemetrexed was given from September 2009 to February 2010. No response changes were observed. Immunotherapy was initiated, and then Pemetrexed was given with the same disease status. Both treatments were well tolerated. Immunotherapy toxicity included reaction at the site of injection grade 2. At present, the patient is still on this treatment. Given the poor prognosis of patients with advanced lung cancer, the combination of both treatments during the stable phase of the disease may improve progression-free survival.

## Case presentation

A 53-years-old woman otherwise previously healthy and a non-smoker, was diagnosed with lung adenocarcinoma, stage IV T 3 Nx M1a (TNM classification 7th edition) on April 2009.

On March 2009, she presented with functional class III dyspnea. A Chest X-ray showed veiling in the left side of the thorax. CAT scans were performed, and a solid lung mass in the left lower lobe, associated with pleural effusion and moderate lung collapse was seen contralateral pleural effusion was also evidenced.

Bronchoscopy showed extrinsic compression of the left lower lobe bronchus. Pleural biopsy by thoracoscopy, and pleurodesis with sclerosing agents were performed (talc).

Histological examination of the pleura revealed a proliferation of epithelial-like atypical cells arranged in glands, nests, and cords with moderate anisocytosis, anisokaryosis, macronucleoli, and scattered mitoses. Pleural fluid was positive for neoplastic cells.

Immunostaining techniques were performed against the following antigens: cytokeratin 7, cytokeratin 20, calrretinina, chromogranin, and TTF, which were positive for cytokeratin 7 and TTF. These morphological findings are related to moderately differentiated adenocarcinoma of pulmonary etiology. Cobas EGFR test was non-mutated.

**Figure 1:**
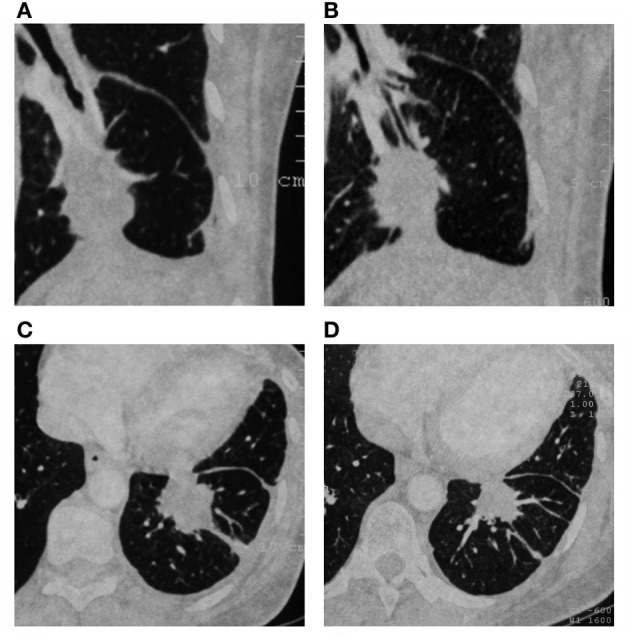
**Stable disease after maintenance treatment with Racotumomab and pemetrexed. (A,C)** Computed tomography in March 2010 before treatment. **(B,D)** Computed tomography in February 2012, last assessment.

First line chemotherapy with carboplatin AUC 6 + paclitaxel 200 mg/m^2^ + bevacizumab 15 mg/kg every 21 days was started on May 2009. The patient received six cycles, and this regimen finished in September 2009. Tumor assessment showed partial response (RECIST). Maintenance therapy with bevacizumab 15 mg/kg + pemetrexed 500 mg/m^2^ every 21 days was administered no significant toxicity was associated with these regimens. Bevacizumab was discontinued in February 2010 and the patient was included in a compassionate program including Racotumomab. Pemetrexed was administered together with immunotherapy, and the patient is still on treatment. Partial response was maintained (Figure 1). As for toxicity associated with the investigational regimen the patient exhibited a reaction at the site of injection of the vaccine grade 2. Adverse events related to pemetrexed were not different from expected, asthenia grade 2.

## Background

Non-small cell lung cancer (NSCLC) is about 85% of all newly diagnosed cases of lung cancer, and the leading cause of cancer related mortality worldwide. Despite some advances in therapy, the overall prognosis is not encouraging yet; as for all stages of this devastating disease, less than 20% of patients are alive 5 years after diagnosis, in the setting of metastatic disease, the median overall survival (OS) is below 1 year and 4–6 months without treatment (Fong et al., 2005; Jemal et al., 2010; Winter et al., 2011).

Conventional therapies for NSCLC such as surgery and radiotherapy are quite effective in the treatment of localized tumors; in the setting of progressive disease, chemotherapy is still the treatment of choice but, because of toxicity involving normal tissue, its use is often limited.

The introduction of first-generation chemotherapy (platinum based regimens including paclitaxel, docetaxel, gemcitabine or vinorelbine) has proven to have only limited activity. The response rate was 10–15%, with slight improvement in OS with median survival rates below 11 months, and 31–36% at 1 year (Winter et al., 2011).

Nowadays, many patients with advanced NSCLC will benefit from the individualized regimens based on the identifiable molecular characteristics of their tumors. The tumor molecular profile should help select the appropriate agents for a given patient (Kim et al., 2012).

One of the proposed treatment algorithms for advanced NSCLC in negative or unknown epidermal growth factor receptor (EGFR) mutation or anaplastic lymphoma kinase fusion protein (EML4-ALK) involves four to six of platinum-based combinations and currently remains one of the preferred approaches in the first-line setting (Schiller et al., 2002; Scagliotti et al., 2008; Gandara et al., 2009).

Second-line therapies have improved OS, but up to 50% of patients completing first-line treatment become ineligible for further treatment, mostly because of significant tumor progression or rapid decline in performance status (PS) ECOG. Therefore, many investigators studied the early use of second-line therapy in the form of maintenance therapy.

Maintenance therapy is defined in the absence of progression after a first-line platinum-containing regimen. Some investigators have studied prolonged platinum partner use from the first-line regimen called “continuation maintenance”; others have studied the use of a non-cross-resistant agent after induction, which has been termed “switch maintenance” (Gridelli et al., 2009a). The approved agents for maintenance therapy include, Pemetrexed, Docetaxel, Erlotinib, Bevacizumab, and Cetuximab. Although some randomized studies have shown a small but significant progression free survival (PFS) and OS benefit for the maintenance treatment, and guidelines recommend some approved drugs as first category, this therapy is not universally considered standard.

Moreover, a better understanding of the immune system regulation is essential, particularly how immune responses against cancer can be induced, which is mainly mediated by an adaptive cellular immune response and finally results in cancer cell recognition and destruction.

New approaches to improve immune responses and treat human malignancies have become increasingly refined. These therapies may prime the immune system to recognize the antigens expressed in tumor cells, but not in normal tissue thus being able to destroy these abnormal cells and leave the normal cells intact. The human immune system uses a complex coordinated set of cells and signaling molecules to either activate or inhibit immune responses to endogenous antigens, which are the most commonly expressed tumor antigens the cellular immune system can use to specifically target cancer cells.

Some preclinical studies have shown that immunotherapy is considerably effective against small tumor burdens, but seems unable to control large masses (Baxevanis et al., 2009). In advanced stage disease, the pre-existing immunity must have been insufficient for tumor eradication, although tumor-specific immune responses have been detected in some cases. This poor immunological response may include acquired or innate host tolerance to tumor-associated antigens, tumor development in an immunoprivileged site, or the expression of tumor-associated proteins suppressing the activity of cytotoxic T lymphocytes, among others.

Therapeutic anti-cancer vaccines are intended to cause or enhance an adaptive immune response to the tumor cells. Therefore, it is important to identify a specific anti-genic stimulus that will be recognized as immunogenic by the patient's immune system, and to create an efficient delivery system to generate a sufficiently good immune response to the antigen leading to a clinically relevant result (Thatcher and Heighway, 2010). In other words, vaccines include a tumor antigen source in order to be immunologically relevant, combined with some type of “adjuvant” to make these tumor antigens more visible to the immune system. It was hypothesized that antibody mediated inflammation could facilitate tumor progression, but high titers of these antibodies may kill tumor cells (Varki, 2010).

For a long time, lung cancer was not considered an immune-sensitive malignancy. However, increasing evidence that NSCLC may evoke specific humoral and cellular anti-tumor immune responses is available. With more knowledge about the link between the induced immune response and a resulting objective clinical response, lung cancer vaccines may be promising in sequence and/or combination with other anti-tumor treatment modalities such as chemotherapy to improve vaccination results (Ma et al., 2004). Both strategies have improved the immune response in both preclinical and human trials.

A number of promising vaccines based on different types of antigenic stimuli have been evaluated in clinical studies; e.g., different immunotherapeutic strategies in lung cancer include, active immunotherapy (vaccines, i.e., MAGE), passive immunotherapy [monoclonal antibodies (mAbs) i.e., Ipilimumab] and adoptive T-cell transfer among others (Kelly et al., 2010).

Gangliosides are a family of sialylated glycolipids that are typical components of the cell membrane. Some of them have been identified as tumor associated antigens capable of inducing an antibody response (Guthmann et al., 2004). For this reason, they are considered possible targets for cancer management, and have become the focus of many immunotherapeutic approaches.

N-acetyl GM3 is one of the most common sialic acid on the cell surface, abundant in normal serum and one of the most immunologically tolerated members of the family. Also, N- glycolyl- GM3 (NGcGM3) is relevant for tumor biology due to its high immunogenicity and expression in several human cancer cells like melanoma, breast and lung cancer but usually not detected in normal tissue. Such features make it an excellent target for immunotherapy (Guthmann et al., 2004; Fernandez et al., 2010; Machado et al., 2011).

Carbohydrate determinants undergo significant changes during malignant transformation. The only structural difference between N- acetyl- GM3 and N- glycolyl- GM3 is a single oxygen atom at the C-5 position of NGcGM3, catalyzed by the cytidinemonophospho-N-acetylneuraminic acid hydroxylase (CMAH). The absence of NeuGc-neuraminic acid in human cells is due to the inactivation of the gene by the enzyme responsible for NeuGc biosynthesis. Evidence suggests that its presence in human cancer might be derived from dietary sources or from an alternative metabolic pathway (Fernandez et al., 2010).

**Figure 2:**
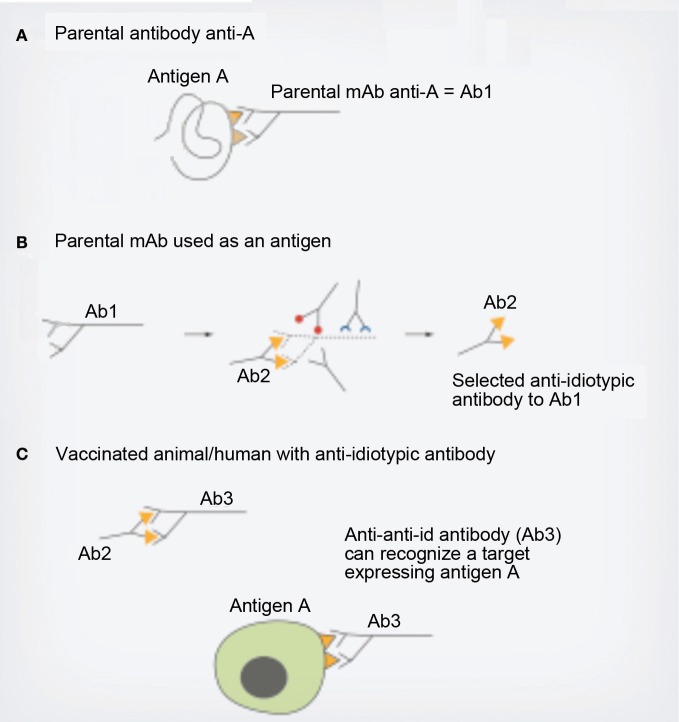
**Anti-idiotypic antibodies.** An anti-idiotypic antibody (Ab2) recognizes the hypervariable idiotypic region of a parental antibody (Ab1). Due to molecular mimicry, an anti-idiotypic antibody may behave like the original antigen A, in particular when the antibody response (Ab3) it triggers when used as a vaccine is similar to the antibody response mounted against the original antigen A (referred to as anti-anti-Id+/anti-antigen+). Immunizing an animal with antigen A **(A)** raises Ab1. Animals immunized with Ab1 will mount a polyclonal antibody response, amongst which Ab2 may be selected as an anti-idiotypic antibody **(B)**. Patients or animals vaccinated with Ab2 may mount an Ab3 antibody response, which may both recognize and kill tumor cells expressing the antigen A **(C)**. mAb, Monoclonal antibody.

One of the approaches to target tumor- associated antigen- expressing cells is the Anti-idiotype vaccination. Anti-idiotypic mAbs have been used as cancer vaccines with encouraging results. This approach comes directly from Jerne's idiotypic network theory. This theory states that due to the huge diversity potentiality of the immunoglobulin variable regions, the idiotype repertoire may mimic the universe of self and foreign epitopes (Machado et al., 2011). Anti-idiotypic mAbs have proved to be able to mimic and induce Ag-specific Ab responses, even against non-protein tumor associated Ags like gangliosides (Figure 2) (Rabu et al., 2012). These anti–anti-idiotypic and anti-ganglioside mAbs may bind to tumor gangliosides and mediate complement-dependent cell lysis or Ab-dependent cell cytotoxicity, inhibit ganglioside-dependent survival cell functions, or block gangliosides release from tumor in patient sera, which are known to have immune suppressive activity (Hernández et al, 2011).

mAbs against gangliosides like NGcGM3 may bind to and mediate anti-proliferative or cytotoxic activities directly against target cells through different mechanisms. Because of the genetic variability and immune evasion capacity of tumors, mAbs with multiple effector mechanisms may be needed to achieve maximal anti-tumor effects.

Racotumomab (formerly known as 1E10) is a vaccine that contains a murine anti-idiotype mAb designed to mimic the NGcGM3 ganglioside. It is an anti-idiotypic IgG (Ab2-type-antibody) generated from the murine tumor model immunization, the F3II mammary carcinoma (BALB/mice), which reacts to the IgM mAb (Ab1-type-antibody), named P3 that recognizes gangliosides as antigens. Both Racotumomab anti-tumoral activity and preclinical toxicity were analyzed in the murine model mentioned above and in the B16 melanoma (C57BL/6 mice). The drug was both safe and effective in these models.

Tumor- specific expression of NGc-containing gangliosides in some human tumors suggests that the induction of an effective immune response against these targets might be useful in patients whose tumors express the antigen. Phase I clinical trials have proven the safety and immunogenicity of Racotumomab in melanoma, breast cancer and small cell lung cancer patients. High titer Ab responses to NeuGc-containing gangliosides were detected in the sera of these patients (van Cruijsen et al., 2009; Fernandez et al., 2010). NGcGM3 is widely expressed in more than 90% of NSCLC (van Cruijsen et al., 2009).

In a phase II compassionate—use study, 71 patients with advanced NSCLC, IIIB, and IV received standard chemoradiotherapy and then received five biweekly injections of 1 mg of Racotumomab intradermically, other 10 doses at 28 days intervals and continued to be immunized at this same time interval if they were in good PS ECOG. The vaccine was well tolerated; no serious adverse events were reported in this patient cohort. The most common adverse event was reaction at the injection site. OS from the time of initial vaccination was 9.93 months (95% CI, 8.61–11.25); 1 year survival rate was 34%, the median survival time of patients who entered the study with partial response or disease stabilization and with PS of 1 was 11.5 months (95% CI, 7.97–15.03 months), considered since the start of vaccination. Those with progressive disease or *PS* = 2 had a median OS of 6.5 months (95% CI, 4.31–8.69 months). A statistically significant correlation was observed between anti-ganglioside response and survival time in a subset of 20 NSCLC patients from this study. Non-responders (*n* = 4) had a median survival time of 6.35 months (95% CI, 4.97–9.67 months), whereas patients who developed IgG and/or IgM antibodies against NGcGM3 had a median survival time of 14.26 months (95% CI, 5.95–17.3 months; *P* < 0.01) (Kelly et al., 2010; Guthmann et al., 2004; Hernández et al., 2008; Gridelli et al., 2009b).

Immune approaches are unlikely to replace conventional cancer therapies but, in combination with other therapies, they may contribute to better results. Moreover, the complex interactions between cancer cells and host elements within the tumor microenvironment imply that targeting one aspect of tumor biology will have clear consequences in other elements involved in both tumor growth and progression.

It is well known that chemotherapy induces cell death by apoptosis. Recent evidence suggests that apoptosis may be highly immunogenic and its immunomodulatory potential is exerted by a variety of mechanisms. For example, chemotherapy may condition the tumor microenvironment by modulating the expression of tumor antigens, accessory molecules of T-cell activation or inhibition, and molecules involved in antigen processing and presentation; furthermore, it may manipulate systemic pathways of immune tolerance and regulation (Emens, 2010).

Some preclinical studies evaluating the combination of vaccines with other oncospecific treatments have been published, providing a rationale for chemoimmunotherapy combinations in the clinical setting (Fernandez et al., 2010). Preclinical models using the 3LL Lewis lung carcinoma in C57BL/6 mice as a model of NSCLC, have shown that the combination of Racotumomab with chemotherapeutics drugs such as Pemetrexed leads to satisfactory results (Segatori et al.).

On the other hand, Racotumomab and low-dose Cyclophosphamide in a mammary carcinoma model significantly reduced breast carcinoma growth in mice, and that response was comparable with the co-administration of the standard high-dose chemotherapy for breast cancer based on 60 mg/m (Winter et al., 2011) of Doxorubicin and 600 mg/m (Winter et al., 2011) of Cyclophosphamide, without toxicity signs (Fuentes et al., 2010).

The Center of Molecular Immunology from Havana, Cuba, where Racotumomab was developed, conducted a phase I study to assess the feasibility of combining the vaccine with the standard first line chemotherapy used in advanced NSCLC. Twenty patients were included and treated with cisplatin/vinblastine. The vaccination schedule was administered concomitantly with chemotherapy and continued beyond progression, until unacceptable toxicity or until the patient decreased PS to grade 3 or lower. Nineteen patients achieved control disease, median survival has not been reached and the mean survival was 12.94 months (Macias et al.).

The combination was considered safe and all the patients developed high antibody responses against Racotumomab during the vaccination schedule as well as IgM and IgG antibody response against NeuGcGm3 antigen as in the standard not concomitant vaccination schedule used in former clinical trials, suggesting that chemotherapy does not inhibit vaccine –mediated immune response (Macias et al.).

## Discussion

In advanced NSCLC, systemic chemotherapy and/or localized irradiation can produce objective responses and palliation of symptoms; however, these therapies are associated with a modest improvement in survival despite continuing advances.

To date, maintenance therapy with either a chemotherapeutic or a molecular target agent after standard first line treatment is one of the strategies that are continuing under investigation in several trials. This strategy has shown a substantially longer progression free survival, but the positive impact on OS is modest. This approach is associated with more frequent adverse events with the consequent impairment on quality of life. For these reasons, this indication is controversial, and is not considered a standard of care in many centers.

Tumor vaccines alone in the treatment of solid tumors had not the expected impact on survival, but its combination with other treatment modalities such as chemotherapy may increase its effectiveness. Evidence suggests that irrespective of the potency of chemotherapy and the specificity achieved with immunotherapy, neither of these by itself has been enough to eradicate the disease. Clinical trials have evaluated the combination of immunotherapy and chemotherapy and have shown synergistic effect between them that could improve the therapeutic efficacy. Certain chemotherapeutic drugs have immunomodulatory activities, enhancing the efficacy of tumor cell vaccines and the immunotherapy response. On the other hand, vaccination may sensitize the tumor to subsequent chemotherapeutic agents and induce a dynamic phenomenon in the host immune response, which it could be modified by concomitant treatment by different ways.

The challenge is to combine both conventional treatment and immunotherapeutic strategies, in order to lower the tumor burden and prepare the host immune system to control minimal residual disease.

Racotumomab has acceptable safety outcomes and is able to induce specific humoral and cellular immune responses. These responses seems to be stronger in those patients with lower tumor burden, better PS and a good response to previous oncological treatments and, in this respect, vaccine therapy might be an appropriate (van Cruijsen et al., 2009).

Regarding Pemetrexed maintenance therapy, the first analysis has shown that Pemetrexed improves median progression-free survival vs. placebo (4.1 months from randomization vs. 2.8 months respectively). This analysis shows an improvement on median OS also (13.9 months from randomization vs. 11 months) (Paz-Ares et al., 2012a,b).

Here, we presented a case report of an advanced NSCLC patient who seems to have been benefited from maintenance therapy and/or immunotherapy. PFS and OS are higher than expected. Toxicities were not higher than described with these agents when used alone.

## Concluding remarks

Given the results presented in this case report we consider that the combination of chemotherapy and immunotherapy deserves further investigation.

### Conflict of interest statement

The authors declare that the research was conducted in the absence of any commercial or financial relationships that could be construed as a potential conflict of interest.
